# Measurements of the Weak UV Absorptions of Isoprene and Acetone at 261–275 nm Using Cavity Ringdown Spectroscopy for Evaluation of a Potential Portable Ringdown Breath Analyzer

**DOI:** 10.3390/s130708170

**Published:** 2013-06-26

**Authors:** Peeyush Sahay, Susan T. Scherrer, Chuji Wang

**Affiliations:** Department of Physics and Astronomy, Mississippi State University, Mississippi State, MS 39762, USA; E-Mails: peeyush.sahay@gmail.com (P.S.); sscherrer@southernionics.com (S.T.S.)

**Keywords:** UV absorption cross-sections, breath biomarker, isoprene, acetone, CRDS

## Abstract

The weak absorption spectra of isoprene and acetone have been measured in the wavelength range of 261–275 nm using cavity ringdown spectroscopy. The measured absorption cross-sections of isoprene in the wavelength region of 261–266 nm range from 3.65 × 10^−21^ cm^2^·molecule^−1^ at 261 nm to 1.42 × 10^−21^ cm^2^·molecule^−1^ at 266 nm; these numbers are in good agreement with the values reported in the literature. In the longer wavelength range of 270–275 nm, however, where attractive applications using a single wavelength compact diode laser operating at 274 nm is located, isoprene has been reported in the literature to have no absorption (too weak to be detected). Small absorption cross-sections of isoprene in this longer wavelength region are measured using cavity ringdown spectroscopy for the first time in this work, *i.e.*, 6.20 × 10^−23^ cm^2^·molecule^−1^ at 275 nm. With the same experimental system, wavelength-dependent absorption cross-sections of acetone have also been measured. Theoretical detection limits of isoprene and comparisons of absorbance of isoprene, acetone, and healthy breath gas in this wavelength region are also discussed.

## 1. Introduction

Noninvasive breath gas analysis for the detection of established biomarkers is rapidly gaining much attention in a variety of scientific and medical communities [1–3]. While the overall process is still in its infancy, much research work is being directed toward the development of portable instruments which offer real-time, fast response, high sensitivity measurements. The traditional approach to gas analysis using gas chromatography-mass spectrometry is hindered by very large, expensive, benchtop instruments which require laborious sample manipulation as well as rigid calibration procedures [4–6]. On the other hand, techniques, such as the proton transfer reaction-mass spectrometry (PTR-MS) and laser spectroscopy, have demonstrated ultra trace detection capability and real-time monitoring of volatile organic compounds (VOCs). PTR-MS based instrument can be designed in a compact configuration and employed for the detection of environmentally important trace gases as well exhaled breath VOCs [7,8]. Similarly, laser-based analysis has made significant strides in the detection and measuring of trace species in a host of environments, and considerable attention has been given to technological advances in the manufacture of lasers for numerous applications over a broad wavelength range [2]. Due in part to the success of the telecommunications diodes, compact diode lasers in a host of wavelengths, such as 266 nm, 274 nm, 1,550 nm, 1,650 nm, *etc.*, are now being manufactured. For breath gas analysis, however, the NIR telecommunications' diode lasers will be significantly hindered in their detection selectivity because the majority of the volatile breath gas constituents have weak absorption at those wavelengths, which typically consists of an asymmetric C–H stretching overtone [9]. In order to selectively differentiate between the various components in the exhaled breath gas using laser-based techniques, one must select a wavelength region(s) in which there is minimal overlap of the absorption spectra of the species of interest as well as with competing spectral interference from atmospheric entities, such as H_2_O, CH_4_, CO_2_, *etc.* [10]. Additionally, detection of trace species is enhanced by selecting wavelengths with strong absorption and or minimum spectral interference from comparatively weak absorption from others, which is often the case for electronic transitions in the ultraviolet (UV) region.

Concurrent with strides in instrumentation, additional effort is placed on the establishment of spectroscopic database, such as wavelength-dependent absorption cross-sections (the spectral fingerprints) for selective identification and accurate quantification of the individual species of interest. The objective of this study was to explore the absorption spectra of two known breath biomarkers, isoprene (2-methyl-1,3-butadiene) and acetone around 266 nm. To this end, measurements of absorption cross-sections of these two biomarkers were conducted in the wavelength range of 261–275 nm, where compact diode lasers can be available [11,12], yet their absorption cross-sections in this wavelength region are either unknown or incomplete. Of the more than 1,000 major volatile organic compounds (VOCs) present in human breath gas, isoprene constitutes one of the few highest abundance VOCs [13–15]. Isoprene is a product of several metabolic processes inside human body and, as Miekisch *et al.* suggested [16], a by-product of cholesterol biosynthesis. It has been recognized as a potential indicator of cholesterol levels in blood [17–19]. Some studies indicate a possible diurnal variation in breath isoprene; however, subsequent research alluded to the fluctuations being associated with an experimental artifact, the manner in which the samples were collected. Isoprene also plays a significant role in atmospheric chemistry in the troposphere. The emission of natural isoprene from non-anthropogenic sources (e.g., deciduous trees and phytoplankton) and combustion can eventually lead to the formation as well as the scavenging of ozone [20–24]. Another important and highly abundant VOC in breath gas is acetone (2-propanone), which is an important breath biomarker, associated with blood glucose concentrations. In our previous studies [25–27], we have investigated the absorption of isoprene and acetone in the NIR spectral region under vacuum conditions and NIR absorption cross-sections were measured. We have also studied breath acetone using a portable cavity ringdown spectroscopy (CRDS) device operating with a single wavelength, palm-size 266 nm laser [27,28]. Breath acetone concentrations in various cases were determined using the absorption cross-section of acetone reported in the literature [10,27,28]. The research goal of this work was to evaluate whether a portable breath analysis device can also be constructed for isoprene using a compact laser in the UV region. Toward this aim, we investigated the wavelength-dependent absorption spectra of isoprene in the wavelength region, which has been reported to have no absorption, using high sensitivity CRDS technique with a tunable optical parametric oscillator (OPO) laser source that can readily reach the UV spectral region.

While the absorption cross-sections of acetone in the 261–275 nm region have been reported [29–31], the absorption cross-sections of isoprene in the wavelength range 261–275 nm is yet to be completed. Few recent measurements on the absorption cross-sections of isoprene at wavelengths longer than 270 nm reported that isoprene has no absorption (too weak to be detected), consequently, isoprene cross-sections in the wavelength longer than 270 nm are not available [32,33]. This spectroscopic data gap hinders the instrument development using a single wavelength diode laser source at 274 nm. In the present study, isoprene absorption cross-sections were measured up to the data gap region (270–275 nm) using high sensitivity OPO-CRDS, the absorption cross-section at 275 nm turned out to be as low as 10^−23^ cm^2^·molecule^−1^ that was not measurable using conventional single-pass absorption spectroscopy techniques. With the same experimental system, CRDS absorption of acetone was also conducted. In addition, ringdown absorption measurements of isoprene, acetone, and 5% carbon dioxide (CO_2_) gas, were conducted as compared to absorption of actual human breath gas.

## 2. Experimental

### 2.1. Cavity Ringdown Spectroscopy

**Figure 1.**
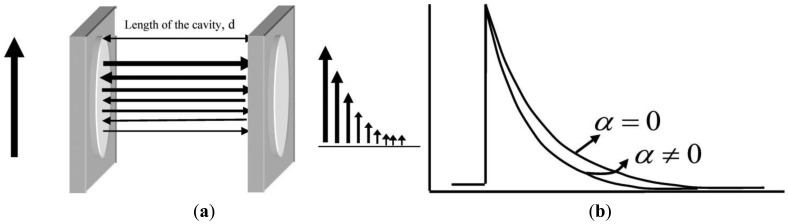
Illustration of the basic CRDS concept. (**a**) Retroflections and the resulting ringdown waveform. (**b**) The induced change in ringdown time as a function of the concentration of the absorbing species present in the beam path.

Cavity ringdown spectroscopy (CRDS), first reported in 1988 by O'Keefe and Deacon, is a powerful laser-based absorption spectroscopic technique [34–36]. CRDS, in its simplest form, is implemented by injecting a laser pulse into a stable optical cavity, consisting of two highly reflective mirrors, as shown in Figure 1. When a laser pulse is incident on the back side of the first mirror, the majority of the pulse is reflected away from the cavity; however, a small portion of the beam, based on the finite reflectivity of the mirror, is injected into the optical cavity. When this injected pulse encounters the second high reflectivity mirror, the majority of the remaining pulse is reflected back into the cavity while a minor percentage is ejected from the cavity. The beam effectively reflects back and forth between the mirror surfaces, transmitting a small percentage of its intensity with each mirror encounter. The transmitted intensity usually follows an exponentially decaying profile, as depicted in the figure. The time duration in which the transmitted intensity decreases to 1/e of the incident intensity injected into the optical cavity is called the ringdown time (*τ*). The ringdown time constant for the exponential decay is given by the following:

(1) $$\tau =\frac{d}{c(1-R+\alpha l_{s})}$$

where *d* is the distance between the mirrors, *c* is the speed of light, *R* is the average reflectivity of the ringdown mirrors, *α* is the wavelength-dependent absorption coefficient, and *l_s_* is the optical pathlength through the sample. For a gas filling the entire cavity, consistent with the research proposed herein, *d* = *l_s_*. When the optical cavity is empty or the wavelength is selected such that there is no absorption by any species present in the cavity (*α* = *0)*, then the empty cavity ringdown time becomes equivalent to *τ* = *d*/*c*(1–*R*). From this expression, one can experimentally determine the average reflectivity of the ringdown mirrors. Once the mirror reflectivity is known, one can determine the concentration of an absorbing species in the cavity if the absorption cross-section is known or, conversely, the absorption cross-section of the species if the concentration of the sample in the cavity is known. Upon introduction of an absorbing species or tuning the laser onto an absorption line of a species in the cavity, *α* increases which directly affects the measured ringdown time. The magnitude of loss (hence the factor by which the ringdown time decreases) depends upon the absorption strength of the sample at the particular wavelength being examined as well as the concentration of the sample in the ringdown cavity and the sample pathlength. From the ringdown time measurement, the absorbance (*A*) is calculated as:

(2) $$A=\frac{d}{c}(\frac{1}{\tau }-\frac{1}{\tau_{0}})=\alpha l_{s}=\sigma (v){\text{nl}}_{s}$$

where σ(*v*)is the wavelength-dependent absorption cross-section of the sample and *n* is the number density of the sample inside the cavity. Thus, for a given ringdown system, the absorption cross-section of the sample can be determined, if the number density is known, and vice versa.

### 2.2. Experimental Set Up

**Figure 2.**
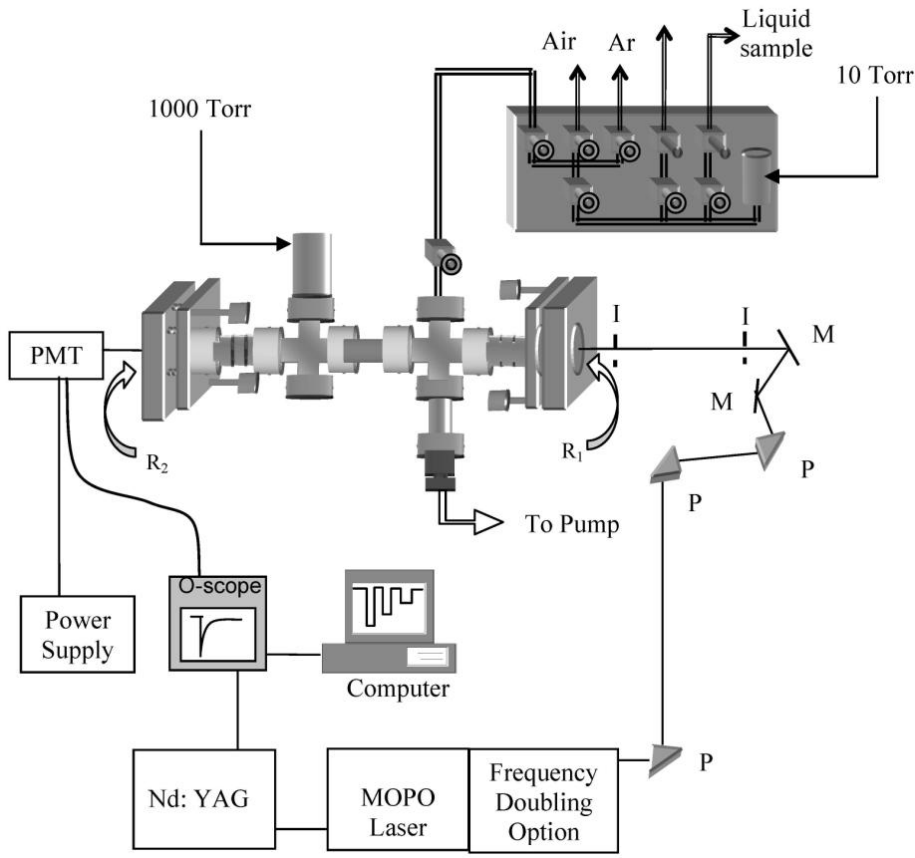
Schematic of the experimental system. This experiment utilized an MOPO-HF-FDO laser system to generate UV laser light, which was injected into a ringdown cavity that was housed in a stainless steel vacuum chamber. An in-house sample introduction system was constructed and utilized. R = ringdown mirror, I = irises, P = UV prism, M = beam steering optics.

The experimental system utilized in this study consists of four primary segments: the laser system and corresponding optics, the detection electronics, a vacuum chamber hosting the ringdown mirrors, and the sample introduction and dilution sections. A schematic of the experimental setup is depicted in Figure 2.

#### 2.2.1. Laser System and Detection Electronics

An OPO (SpectraPhysics, MOPO-FDO-HF) pumped by an Nd:YAG (Qaunta Ray Pro-290, SpectraPhysics) was employed to generate the wavelengths in the UV. The 355 nm output of the YAG was injected into the MOPO and subsequently frequency doubled to the UV wavelengths (261–275 nm) examined in this study. The MOPO system was outfitted with the high finesse frequency doubling option to allow 0.001 nm wavelength resolution. The output beam was directed through a series of prisms, beam steering optics, and irises prior to entering the ringdown cavity to ensure wavelength separation due to collinear visible output and stray 355 nm transmissions. The signal exiting the ringdown cavity was detected by a PMT (Hamamatsu, R928) placed behind the second ringdown mirror. A Tektronix TDS 410A oscilloscope, interfaced to a PC, captured each ringdown event. An in-house fabricated ringdown program determined the ringdown time corresponding to each ringdown waveform provided by the oscilloscope. Upon completion of the preliminary data acquisition, a UV pulsed wavemeter (Burleigh/EXFO, WA-5500) was incorporated to verify the output wavelength of the MOPO system.

#### 2.2.2. Sample Introduction and the Vacuum Chamber

Absorption measurements of each sample were made at atmospheric pressure by introducing a known quantity (generally a few Torr) of the sample into the vacuum chamber and then bringing the total pressure to the atmospheric pressure with ultra-high purity argon (>99.99%, Airgas). Isoprene in N_2_ (1% by weight (99.99% purity)) and acetone in N_2_ (0.8% by weight (99.99% purity)) were obtained from Scott Specialty Gases. The vacuum chamber hosted two high reflectivity ringdown mirrors (Los Gatos Research), R = 99.8%, connections for the sample introduction, the pumping mechanism, and multiple pressure transducers. The ringdown mirrors were mounted in the ends of the stainless steel vacuum chamber in Gimbal mounts, which were connected to the chamber via flexible bellows, and spaced 74 cm apart. A rotary pump was employed to evacuate the chamber between samples and aid in sample dilutions. A 10 Torr MKS pressure transducer was utilized to accurately and reproducibly introduce measurable amounts of gaseous samples into the ringdown cavity, and a second gauge (1,000 Torr, MKS) allowed the chamber to be taken to atmospheric pressure with argon. Sequential dilutions were performed by partially evacuating the ringdown chamber with the rotary pump and filling the chamber to atmospheric pressure (set at 760 Torr) with the argon. At each dilution, the corresponding ringdown time was obtained. This procedure was implemented for all isoprene and acetone data in this study. To eliminate any measurement deviation due to temperature effects, the room temperature was maintained at 296 ± 2 K throughout the experiment. The ringdown time recorded at vacuum was approximately 1.40 μs. The stability of the signals is measured by the baseline stability, which is defined as $\frac{\sigma_{\tau }}{\bar{\tau }}$, where *σ_τ_* is the standard deviation of the ringdown signals and *τ̅* is the average ringdown time. Typically, the baseline stability for the vacuum chamber throughout the experiments remained to be ≤0.5%.

## 3. Results and Discussion

### 3.1. UV Absorption Cross-Sections of Isoprene in the Wavelength Range of 261-269 nm

**Figure 3.**
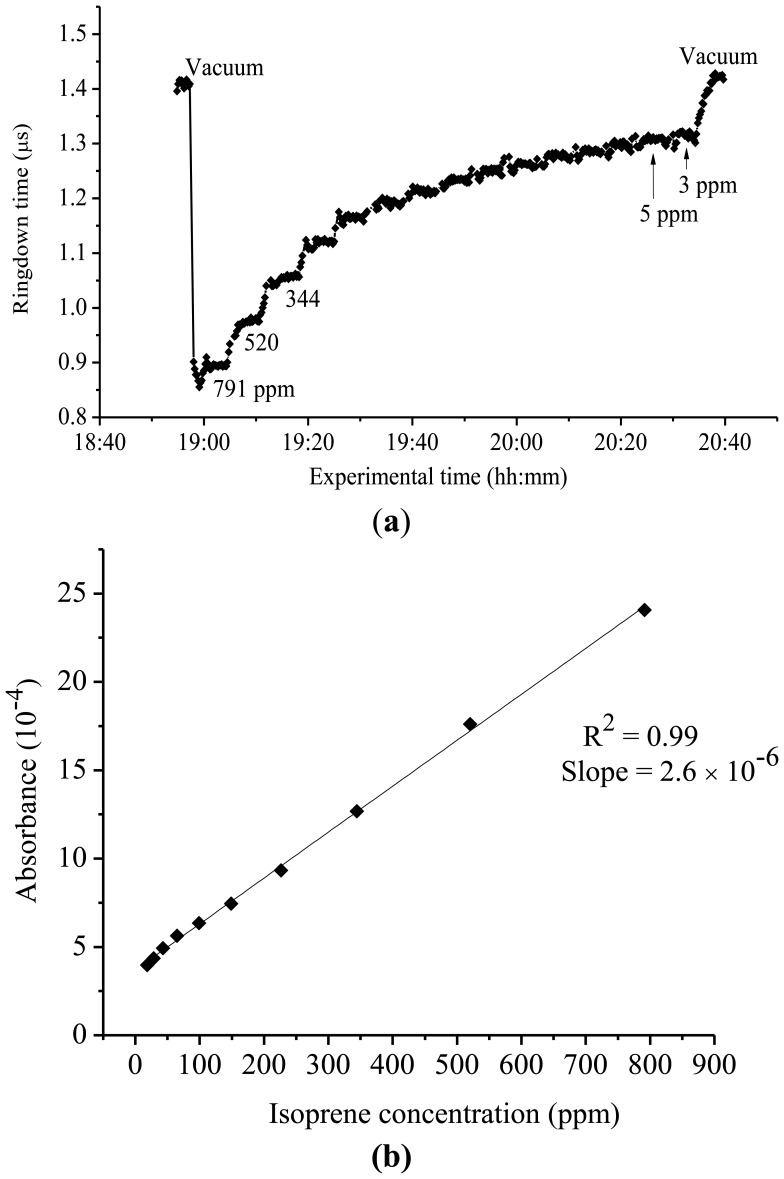
(**a**) Ringdown absorption graph for isoprene at 266 nm (**b**) Corresponding curve of the absorbance *versus* concentration.

Since isoprene has been identified as a potential biomarker associated with cholesterol synthesis, particular interest has been placed on accurately detecting and measuring its presence, especially in breath gas [32,33,37-40]. At 266 nm, there is a distinction between the UV spectra of isoprene and pentane. However, there is a discrepancy in the reported literature as to the absorption cross-section of isoprene and the ability to obtain this value in the mid-UV region. However, using the highly sensitive CRDS approach, the presence of isoprene in this region is detectable. Figure 3(a) shows a typical ringdown curve of isoprene at 266 nm. Figure 3(b) shows the experimental curve of the absorbance of isoprene at 266 nm *versus* its concentration. Each data point shown in Figure 3(a) was generated by averaging over 100 ringdown events. At this fixed wavelength, the ringdown times were recorded in the isoprene concentration range of 3-791 ppm. The steps in the graph represent individual concentrations of isoprene. As it can be noticed in the graph, a significant change in ringdown time, from 1.4 μs to 0.88 μs, was observed when a 791 ppm isoprene sample was introduced into the vacuum chamber. In the lower concentration region, *i.e.* at 3 ppm and 5 ppm, which were close to the detection limit of 4 ppm for isoprene at 266 nm with the present CRDS system, the ringdown signals were noisy (the baseline stability >1%). Therefore, no measurement was conducted at isoprene concentrations lower than 3 ppm. Good linearity (R^2^ = 0.994), as shown in Figure 3(b), was routinely obtained at each wavelength examined for isoprene. Using this approach, the absorption cross-section for isoprene at 266 nm was determined to be 1.42 × 10^−21^ cm^2^·molecule^−1^. The same experiment was repeated at 261 nm wavelength. A cross-section of 3.65 × 10^−21^ cm^2^·molecule^−1^ was determined for isoprene at 261 nm.

### 3.2. New UV Absorption Cross-Sections of Isoprene in the 270–275 nm

The studies utilizing gas chromatography coupled with UV detection [41] as well as high resolution VUV absorption spectroscopy [33] reported that isoprene has no absorption or the absorption is too weak to be detected in the wavelength region longer than 269 nm [33]. Therefore, along with the ringdown measurements of isoprene at 261 and 266 nm, the absorption spectra of isoprene in the wavelength region of 270-275 nm were also investigated, in particular, measuring its absorption cross-sections at two specific wavelengths, 270 nm and 275 nm.

**Figure 4.**
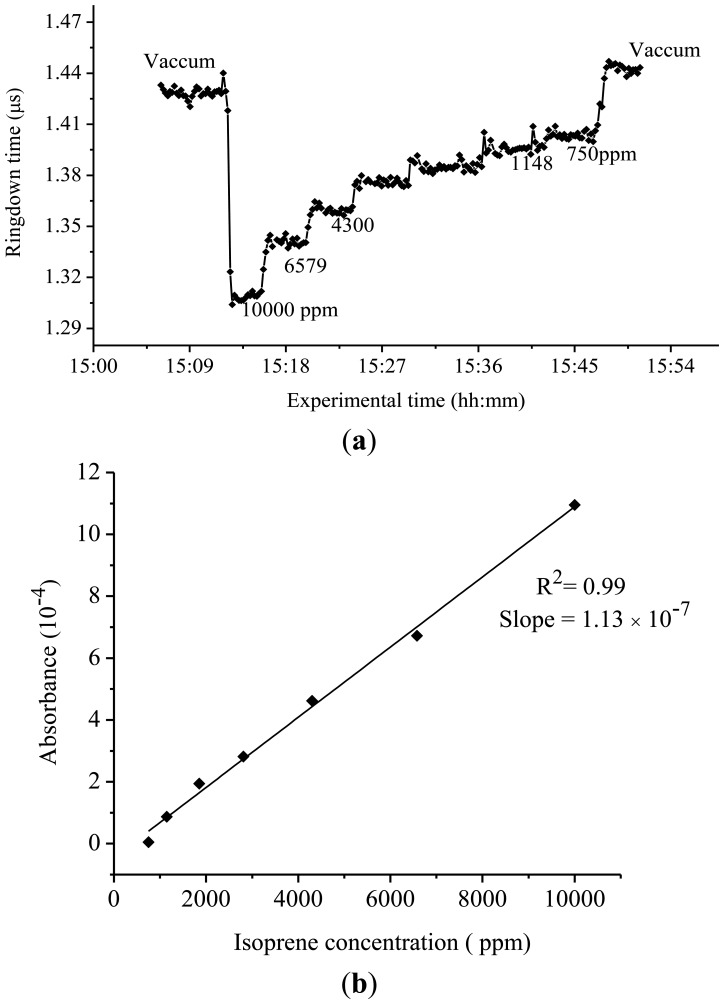
(**a**) Ringdown absorption graph for isoprene at 275 nm (**b**) Corresponding curve of the absorbance *versus* concentration.

The results of the ringdown measurements of isoprene conducted at 275 nm are shown in Figure 4. Owing to the very low absorption strength in this region (confirmed later by the results of this experiment), a concentration of isoprene gas samples up to a few thousands of ppm was required in order to observe a significant change in the ringdown time. For example, at the 275 nm wavelength, when 10,000 ppm isoprene gas sample was introduced into the chamber, the ringdown time dropped to 1.30 μs from 1.42 μs in the vacuumed chamber, as shown in the Figure 4(a). Subsequently, the ringdown time increased only up to 1.33 μs even when the isoprene concentration in the chamber was decreased to 6,579 ppm. The difference in the ringdown times narrowed down further with decrease in the isoprene concentration. At 1,148 and 750 ppm, the difference in the ringdown time was only 0.01 μs; the actual ringdown times recorded at these concentrations were 1.39 μs and 1.40 μs, respectively. Figure 4(b) shows the experimental curve of the measured isoprene absorbance at 275 nm *versus* isoprene concentration. A good linearity, R^2^ = 0.99, was obtained. Based on the slope of the curve, the absorption cross-section of isoprene at 275 nm was measured to be 6.20 × 10^−23^ cm^2^·molecule^−1^. Similarly, absorption cross-section of isoprene at 270 nm was also determined, which was 4.81 × 10^−22^ cm^2^·molecule^−1^.

Table 1 lists the absorption cross-sections of isoprene measured in the wavelength range 261–275 nm in this work and those reported in the literature in the wavelength range 261–269 nm. The cross-sections measured at 261 and 266 nm are in accordance with those reported in the literature, while the cross-sections at 270 and 275 nm are the new measurements.

### 3.3. Discussion on the Weak Absorption Profile of Isoprene in the Wavelength Region 261–275 nm

**Figure 5.**
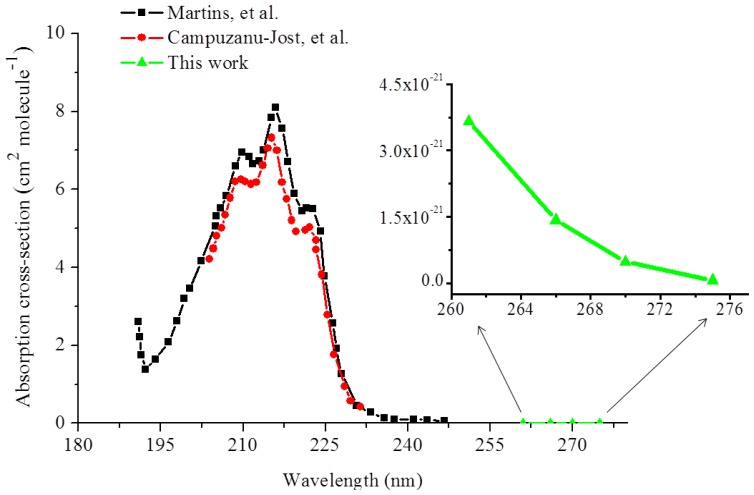
UV absorption profile of isoprene obtained in this work along with the data reported in the literature. The curve denoted by dots shown in the inset is from this work.

According to the spectra of isoprene in the deep UV as reported in the literature [32,33], isoprene has an absorption peak at 215 nm and tapers considerably off toward the mid-UV region. In the high resolution measurements of isoprene presented by Campuzanu-Jost, *et al.* [32] utilizing pulsed laser photolysis-pulsed laser induced fluorescence, the absorption cross-sections of isoprene from 205 nm to 233 nm were reported. Their values are in excellent agreement with those reported by Jones, *et al.* [44], who examined pure isoprene in standard solutions and isoprene in breath gas, and by Martins, *et al.* [33] for the same spectral region. Their measured absorption cross-sections are plotted by the circle and square curves in the wavelength lower than 250 nm in Figure 5. Based on the absorption profiles, one may intuitively infer that the contour of the isoprene absorption spectrum rapidly taper down in the longer wavelength region and the absorption in the longer wavelength end (>250 nm) is too weak to be measured. Figure 5 also shows the absorption cross-sections (the triangle curve) in the wavelength range of 261–275 nm measured in this work. The cross-section values which we have obtained from 261 nm to 275 nm readily exemplify the aforementioned rationale.

As can be seen in the Figure 5 as well as in Table 1, the cross-sections determined at 270 and 275 nm in this work are at least one order of magnitude smaller than the absorption cross-sections at 266 nm, *i.e.*, 4.81 × 10^−22^ cm^2^·molecule^−1^ at 270 and 6.20 × 10^−23^ cm^2^·molecule^−1^ at 275 nm, which are several orders of magnitude smaller than the ones reported in the even shorter wavelength region, *i.e.*, <250 nm. This significant decrease in the absorption cross-section of isoprene from 261 to 275 nm is the manifestation of the overlook of the weak isoprene absorption in this wavelength region.

### 3.4. Theoretical Detection Limits of Isoprene at 266 and 275 nm

In CRDS experiments, the detection limit (DL), *i.e.*, the minimum concentration of a sample that can be determined, depends on the minimum absorbance that a particular CRDS system can measure. The minimum absorbance, *A_min_*, is determined by the baseline stability of ringdown signal and the reflectivity of the mirrors

(3) $$A_{\min}=\frac{\sigma_{\tau }}{\bar{\tau }}(1-R)=n_{\min}\sigma_{\text{abs}}(v)l_{s}$$

where $\frac{\sigma_{\tau }}{\bar{\tau }}$ is the baseline stability. *n_min_* is the minimum detectable concentration. *σ_abs_(ν)* is absorption cross-section of the sample molecule at frequency *ν. R, c*, and *l_s_* are defined in Equation (1). In this case, the path length is equal to the length of the cavity. Subsequently, the 1-σ detection limit is determined as:

(4) $$\text{DL}=n_{\min}=\frac{A_{\min}}{\sigma_{\text{abs}}(v)l_{s}}$$

From Equations (3) and (4), it is clear that DL can be decreased by increasing the reflectivity of the mirror and path length of the laser beam inside the cavity. Considering the mirror reflectivity 99.99%, the detection limits for isoprene were calculated at all the four wavelengths used in this work for a cavity length of 74 cm. The estimated DLs are listed in Table 2. In accordance with the decrease in the absorption line intensity of isoprene with increase in the wavelength from 261 to 275 nm, DL also increases proportionally. The DLs at 261 and 275 nm were determined to be 0.045 and 2.65 ppm, respectively. Similarly, at 266 nm, where palm size diode lasers are available, DL is determined to be 0.12 ppm (=120 parts per billion (ppb)). This result suggests that if mirrors of reflectivity 99.99% are used, then using the CRDS technique at 266 nm, isoprene concentrations as low as sub ppm levels can be measured. Table 2 also lists the DL of isoprene at all the four wavelengths with the present CRDS system.

### 3.5. UV Absorption Cross-Sections of Acetone

Absorption cross-sections of acetone around 266 nm were also investigated with the same experimental system. A few milli Torr of acetone gas when diluted to 760 Torr in Ar, corresponding to acetone concentrations higher than 100 ppm, was introduced into the chamber. Subsequently, the ringdown times were measured. Afterwards, concentrations of acetone in the mixture were reduced in steps eventually to as low as 0.2 ppm; the ringdown times were recorded in each of the steps. A calibration curve of absorbance *versus* concentration was constructed.

**Figure 6.**
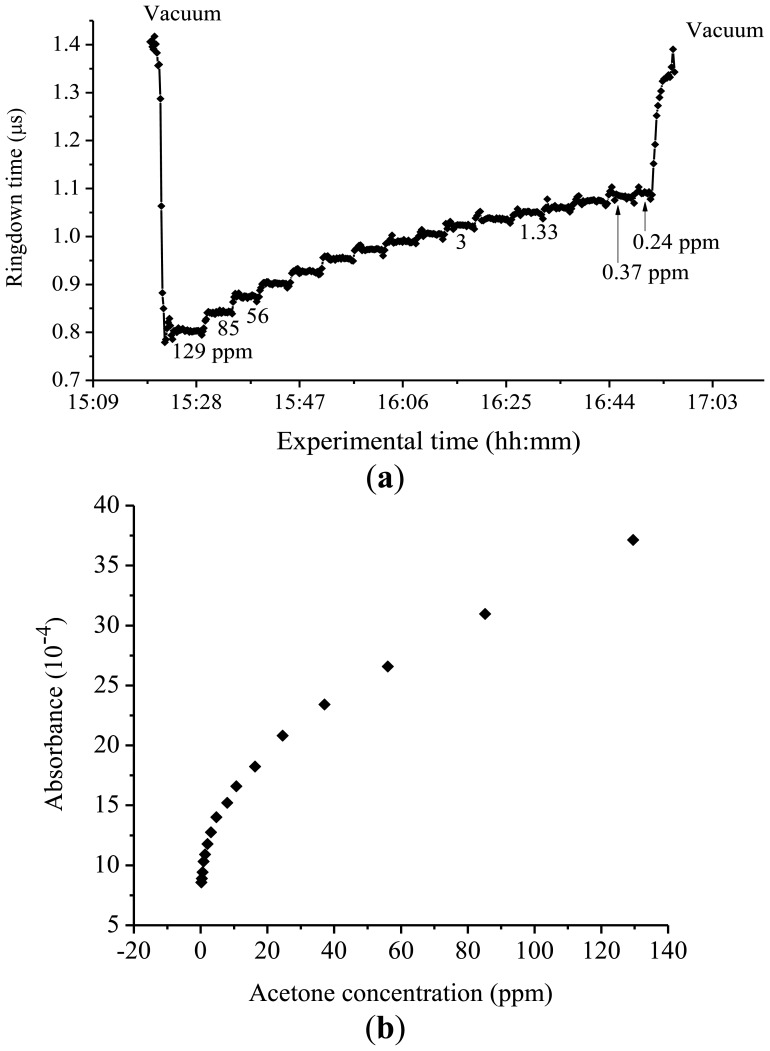
(**a**) Ringdown absorption graph for acetone at 266 nm (**b**) Corresponding curve of the absorbance *versus* concentration.

Absorption cross-sections of acetone at 266 nm have been previously reported using CRDS in conjunction with a compact 266 nm pulsed laser of 1–3 μJ per pulse. In that study, excellent linearity was obtained in the curve of acetone absorbance *versus* acetone concentration and the absorption cross-section of acetone was determined to be 4.5 × 10^−20^ cm^2^·molecule^−1^, which was in agreement with the literature values [29–31]. In this work, however, the absorbance *versus* concentration plot generated a nonlinear curve, as shown in Figure 6.

Using the same experimental system, measurement parameters and procedures, we did not observe this phenomenon in the ringdown measurements of isoprene or in the previous ringdown measurements of acetone at 266 nm. Multiple wavelengths around 266 nm were explored, and the same trend was consistently observed for all of the acetone studies conducted in this wavelength region. Upon a closer inspection of the plots, one can find that the experimental curve can be divided into three concentration regions, namely, <1 ppm, 1–30 ppm, and >30 ppm, while a slow bend appears in the concentration range of 1–30 ppm. Therefore, the absorption cross-section of the acetone could not be determined from a calibration curve, *i.e.*, absorbance *versus* concentration graph, using all of the concentrations of acetone measured. In the lowest concentration range, <1 ppm, the measurements were not accurate enough to determine absorption cross-section due to the large uncertainty in handling the low concentration gas samples. In the highest concentration range, >30 ppm, the data point showed linear behavior as against acetone concentration; however, non-single experiential behavior in ringdown signals was observed. Apparently, a reliable cross-section could not be derived from the data measured in this concentration range. In the concentration range of 1–30 ppm, only when the concentration region of 1–9 ppm was used to plot a calibration curve, the slope of that concentration region gave a value close to the cross-section of acetone reported in the literature. This is because that the acetone absorption cross-section at 266 nm is well known. Otherwise, this unexplained bending curve does not allow one to obtain a reliable value of cross-section. As mentioned earlier, this non-linear behavior between the absorbance and concentration of acetone in the mid-UV was consistently reproducible. At 266 nm, the baseline stabilities for the lower and upper concentrations consistently remained at ≤ 0.5%, except for at 0.24 ppm, the lowest acetone concentration measured, where the baseline stability was 0.7%. The reason for the comparatively high baseline noise at 0.24 ppm can be attributed to the approaching detection limit of acetone measurement at 266 nm. With the present CRDS system, *i.e.*, R = 99.8% and the cavity length of 74 cm, the detection limit for acetone at 266 nm is 0.12 ppm.

In addition to the consideration of the experimental errors resulting from the non-single exponential behavior in the high concentration region or from the handling of low concentration samples (<1 ppm), additional attention was paid to the possible origins of the nonlinear effect, such as possibilities of wall effect, photodissociation of acetone molecules, *etc.* Note that, the α-cleavage of acetone to form acetyl and methyl radicals can be induced with an energy of 81 kcal/mole (3.51 eV/molecule) [45-47]. Therefore, all of these possibilities should be considered while conducting similar studies in the wavelength region in which this study was conducted, specifically, 4.50–4.75 eV. Detailed discussion on the photochemistry of acetone in this UV spectral region can be seen in the literature [45-48] and is not covered in this work.

## 4. Comparison of Ringdown Measurements of Actual Breath Gas with the Pure Acetone, Isoprene and CO_2_

With regard to breath analysis, the present study was further extended to investigate the possible interference to the ringdown absorption measurement of breath acetone from different VOCs, such as isoprene and carbon dioxide (CO_2_), which are present in high abundance in exhaled breaths. Responses of the CRDS system to acetone, isoprene, and CO_2_ in terms of ringdown measurements at 266 nm were compared. Further, the results were also compared with ringdown measurements of actual human exhaled breath gas.

**Figure 7.**
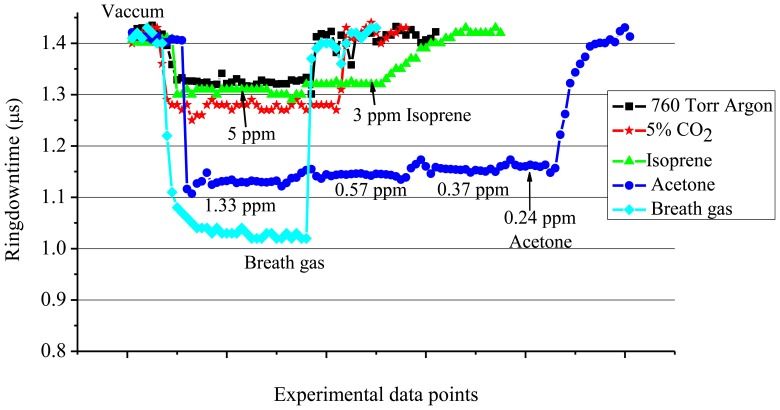
Comparison of ringdown absorption measurements of acetone, CO_2_, isoprene, and actual human exhaled breath gas.

From various experimental results obtained in the present work, the ringdown absorption data were taken in the concentration range of 3-5 ppm for isoprene, in the range of 0.24-1.33 ppm for acetone, and at 760 Torr for 5% CO_2_ in argon gas by volume. Their ringdown absorption results are presented in Figure 7. The ringdown times with the vacuum cavity or the cavity filled with 760 Torr Ar were consistently at 1.40 and 1.32 μs, respectively. The decrease in the ringdown time upon introducing 760 Torr Ar into the chamber is attributed to the scattering losses of the light by Ar atoms, as the absorption strength of Ar at 266 nm is negligible. In either case, the ringdown signal stability remained the same, 0.5%. In the subsequent text, the ringdown time obtained in the vacuum, *i.e.*, 1.40 μs, will be termed as “the vacuum baseline”, and similarly “the Ar baseline” means the ringdown time of 1.32 μs when the chamber was filled with pure argon gas to 760 Torr.

When 5% CO_2_ in Ar was injected into the chamber, the vacuum baseline shifted from 1.40 μs to 1.30 μs. However, when compared with the argon baseline, 1.32 μs, this change is only of 0.02 μs. Since CO_2_ molecules have minimal absorption at the 266 nm, the decrease in the ringdown time from the vacuum baseline is mainly due to the scattering losses of the laser beam. A similar scenario was observed for isoprene. In the numerous experiments conducted for isoprene at 266 nm, it was repeatedly observed that when the concentrations of isoprene were decreased to the level of 3-5 ppm, the ringdown time became close to the Ar baseline. The ringdown times obtained at the isoprene concentrations of 3 and 5 ppm were 1.31 and 1.30 μs, respectively. On the other hand, the measurements of acetone showed that even 1 ppm acetone had a strong absorption. As shown in Figure 7, the recorded ringdown time at 1.33 ppm acetone was 1.12 μs. Subsequently, stepwise increase in the ringdown time with decreasing acetone concentration was recorded. The change in the ringdown time became negligible when the acetone concentration changed from 0.37 ppm to 0.24 ppm, since the concentration approached to the theoretical DL of acetone, 0.12 ppm.

Furthermore, the absorption cross-section of acetone at 266 nm is approximately 32 times larger than the absorption cross-section of isoprene at 266 nm, consequently, the reason for such a huge difference in the ringdown absorption spectra of acetone and isoprene, shown in Figure 7, can be attributed to the much stronger absorption strength of acetone at 266 nm. This experiment demonstrated that for the same level of concentrations of isoprene and acetone, the acetone exhibits much stronger absorption in the CRDS experiment at 266 nm. Therefore, the results suggest that the CRDS technique at 266 nm can be used to measure acetone in an acetone-isoprene mixture without any significant interference caused by isoprene, if both are present in the same level of concentrations.

In the final part of this study, the results were compared with actual breath gas. Breath gas from a healthy human subject was collected in a Tedlar bag and injected into the chamber. Subsequently, the ringdown times were recorded. As depicted in Figure 7, upon injecting the breath gas inside the chamber, the vacuum baseline (1.40 μs) dropped to 1.02 μs. Ringdown times were recorded continuously for a few minutes while the stability of the signal remained at 0.5%. In this particular experiment, the drop in the ringdown signal when the breath gas was injected inside the chamber was slightly larger than the drop in the ringdown signal when a 1.33 ppm acetone gas was measured. The acetone concentration in healthy human exhaled breath varies from 0.1-2.7 ppm [5,14,49,50], while isoprene has been reported to be in the range of few hundreds of ppb [14-18,43,50,51]; therefore, this result implies that there is a possibility of the contributions from other VOCs present in the exhaled breath. In general, most of the VOCs in breath gas are present in very low concentration as compared to acetone, isoprene, and CO_2_ (CO_2_ is about 5% in normal human breath gas). In addition, most of the VOCs in breath gas do not have strong absorption at 266 nm. Therefore, other than acetone, no single VOC present in the breath gas alone can cause such a large difference in the ringdown time. However, a number of VOCs in terms of absorption as well as scattering do have an ineligible amount of collective contribution to the change in ringdown signals, which needs to be considered in the development of a data processing algorithm when breath gas is measured at 266 nm.

## 5. Conclusions

Examination of exhaled human breath gas for biomarkers using a laser-based instrument introduces many favorable detection characteristics which are not available with conventional detection methods, such as simplified sample collection and negligible user inconvenience, *i.e.*, no fingerpricks or urine samples. The absorption spectra of isoprene and acetone were investigated in the mid-UV spectral region, where compact laser sources, *i.e.* single wavelength diode lasers at 266 nm and 274 nm, can be available for the construction of a portable device. Weak absorption cross-sections of isoprene in the wavelength region 261-275 nm were determined to be from 3.65 × 10^−21^ cm^2^·molecule^−1^ at 261 nm to 6.20 × 10^−23^ cm^2^·molecule^−1^ at 275 nm. The absorption cross-section of isoprene determined at 266 nm, 1.42 × 10^−21^ cm^2^·molecule^−1^, is approximately 32 times smaller than the reported absorption cross-section of acetone at 266 nm, *i.e.* 4.5 × 10^−21^ cm^2^·molecule^−1^. The extremely small absorption cross-sections of isoprene at 270 nm and 275 nm, once reported to have no absorption, were determined for the first time in this work. Possibilities of CRDS-based instrumentation for isoprene detection using a 266 nm diode laser and acetone detection using a 274 nm diode laser have been discussed. A comparison of ringdown measurement of actual breath gas with the pure acetone, isoprene, and CO_2_ at 266 nm was also conducted; the result indicated that the ringdown absorption shown by the breath gas has major contribution coming from the bRingdown absorption graph for acetone at reath acetone only. Further, since the absorption cross-section of isoprene measured at 275 nm turned out to be 2 orders of magnitude smaller the absorption cross-section of acetone at 274 nm, it suggests that isoprene will generate minimal interference to the acetone measurement at 274 nm. Therefore, given that small diode lasers at 274 nm can be available, the 274 nm can possibly be a better choice for the development of a small size instrument for acetone measurement. Although detection of acetone can be achieved using a compact diode laser at 266 nm, relatively strong absorption of isoprene at 266 nm needs to be taken care in data processing algorithms.

## Figures and Tables

**Table 1.** Absorption cross-sections of isoprene around 266 nm: A comparison of the data from this work with those reported in the literature.

**Wavelength (nm)**	**Absorption Cross-Section by Mui & Grunwald (cm^2^ ·molecule^−1^) [42,43]**	**Absorption Cross-Section by Martins, *et al.* (cm^2^·molecule^−1^) [33,43]**	**Absorption Cross-Section in this work (cm^2^ ·molecule^−1^)**
258	2.4 × 10^−20^	2.5 × 10^−20^	
260	1.49 × 10^−20^	1.67 × 10^−20^	
261		9.5 × 10^−21^	(3.65 ± 0.18) × 10^−21^
262	8.41 × 10^−21^	5.60 × 10^−21^	
264	5.73 × 10^−21^	6.15 × 10^−21^	
266	2.29 × 10^−21^		(1.42 ± 0.07) × 10^−21^
268	1.53 × 10^−21^		
269			
270			(4.81 ± 0.24) × 10^−22^
275			(6.20 ± 0.31) × 10^−23^

**Table 2.** Detection limits of isoprene with the present CRDS system with mirror reflectivities R = 99.99 and 99.8%.

**Wavelength (nm)**	**Absorption Cross-Section (cm^2^·molecule^−1^)**	**Detection Limit (ppm) R = 99.99%**	**Detection Limit (ppm) R = 99.8% (This work)**
261	3.65 × 10^−21^	0.045	1.5
266	1.42 × 10^−21^	0.12	3.86
270	4.81 × 10^−22^	0.34	11.4
275	6.20 × 10^−23^	2.65	88.6

## References

[1] Mui P.W., Grunwald E. (1984). Enthalpy change for the s-trans to cis-trans conformational equilibrium in 2-methyl-1,3-butadiene (isoprene), as studied by high-temperature ultraviolet absorption spectroscopy. J. Phys. Chem..

[2] MPI-Mainz-UV-VIS Spectral Atlas of Gaseous Molecules.

[3] Mürtz M. (2005). Breath diagnostics using laser spectroscopy. Opt. Photon. News.

[4] McCurdy M.R., Bakhirkin Y., Wysocki G., Lewicki R., Tittel R.F.K. (2007). Recent advances of laser-spectroscopy-based techniques for applications in breath analysis. J. Breath Res..

[5] Wang C., Sahay P. (2009). Breath analysis using laser spectroscopic techniques: Breath biomarkers, spectral fingerprints, and detection limits. Sensors.

[6] Buszewski B., Kesy M., Ligor T., Amann A. (2007). Human exhaled air analytics: Biomarkers of diseases. Biomed. Chromatogr BMC.

[7] Grote C., Pawliszyn J. (1997). Solid-phase microextraction for the analysis of human breath. Anal. Chem..

[8] Lord H., Yu Y.F., Segal A., Pawliszyn J. (2002). Breath analysis and monitoring by membrane extraction with sorbent interface. Anal. Chem..

[9] Blake R.S., Monks P.S., Ellis A.M. (2009). Proton-Transfer reaction mass spectrometry. Chem. Rev..

[10] Herbig J., Amann A. (2009). Proton transfer reaction-mass spectrometry applications in medical research. J. Breath Res..

[11] Scherrer S.T., Wang C., Winstead C.B. Near Infrared Measurements of Volatile Organic Compounds Using Diode Laser Cavity Ringdown Spectroscopy.

[12] Wang C., Mbi A. (2007). A new acetone detection device using cavity ringdown spectroscopy at 266 nm: Evaluation of the instrument performance using acetone sample solutions. Meas. Sci. Technol..

[13] Semiconductor Lasers: Laser diodes are getting the green light. Laser Focus World. http://www.laserfocusworld.com/articles/print/volume-45/issue-4/world-news/semiconductor-lasers-laser-diodes-are-getting-the-green-light.html.

[14] Nomura I., Kishino K., Ebisawa T., Sawafuji Y., Ujihara R., Tasai K., Nakamura H., Asatsuma T., Nakajima H. (2009). Photopumped green lasing on BeZnSeTe double heterostructures grown on InP substrates. Appl. Phys. Lett..

[15] Pauling L., Robinson A.B., Teranishi R., Cary P. (1971). Quantitative analysis of urine vapor and breath by gas-liquid partition chromatography. Proc. Natl. Acad. Sci. USA.

[16] Jansson B.O., Larson B.T. (1969). Analysis of organic compounds in human breath by gas chromatography-mass spectrometry. J. Lab. Clin. Med..

[17] Mendis S., Sobotka P.A., Euler D.E. (1994). Pentane and isoprene in expired air from humans: Gas-chromatographic analysis of single breath. Clin. Chem..

[18] Miekisch W., Schubert J.K., Noeldge-Schomburg G.F.E. (2004). Diagnostic potential of breath analysis—focus on volatile organic compounds. Clin. Chim. Acta.

[19] Kohlmuller D., Kochen W. (1993). Is n-pentane really an index of lipid peroxidation in humans and animals? A methodological reevaluation. Anal. Biochem..

[20] Taucher J., Hansel A., Jordan A., Fall R., Futrell J.H., Lindinger W. (1997). Detection of isoprene in expired air from human subjects using proton-transfer-reaction mass spectrometry. Rapid Commun. Mass Spectrom..

[21] Stone B.G., Besse T.J., Duane W.C., Evans C.D., de Master E.G. (1993). Effect of regulating cholesterol biosynthesis on breath isoprene excretion in men. Lipid.

[22] Guenther A., Hewitt C.N., Erickson D., Fall R., Geron C., Graedel T., Harley P., Klinger L., Lerdau M., McKay W.A., Pierce T., Scholes B., Steinbrecher R., Tallamraju R., Taylor J., Zimmerman P. (1995). A global model of natural volatile organic compound emissions. J. Geophys. Res..

[23] McKay W.A., Turner M.F., Jones B.M.R., Halliwell C.M. (1996). Emissions of hydrocarbons from marine phytoplankton-some results from controlled laboratory experiments. Atmos. Res..

[24] Gil-Av E., Shabtai J. (1963). Precursors of carcinogenic hydrocarbons in tobacco smoke. Nature.

[25] Hauglustaine D.A., Madronich S., Ridley B.A., Flocke S.J., Cantrell C.A., Eisele F.L., Shetter R.E., Tanner D.J., Ginoux P., Atlas E.L. (1999). Photochemistry and budget of ozone during the Mauna Loa Observatory Photochemistry Experiment (MLOPEX 2). J. Geophys. Res..

[26] Fuentes J.D., Lerdau M., Atkinson R., Baldocchi D., Bottenheim J.W., Ciccioli P., Lamb B., Geron C., Gu L., Guenther A., Sharkey T.D., Stockwell W. (2000). Biogenic hydrocarbons in the atmospheric boundary layer: A review. Bull. Am. Meteorol. Soc..

[27] Wang C., Scherrer S.T., Hossain D. (2004). Measurements of cavity ringdown spectroscopy of acetone in the ultraviolet and near-infrared spectral regions: Potential for development of a breath analyzer. Appl. Spectrosc..

[28] Cias P., Wang C., Dibble T. (2007). Absorption cross-section of the C–H overtone of volatile organic compounds: 2 methyl-1, 3-butadiene (isoprene), 1, 3-butadiene, and 2, 3-dimethyl-1, 3 butadiene. Appl. Spectrosc..

[29] Wang C., Mbi A., Shepherd M. (2010). A study on breath acetone in diabetic patients using a cavity ringdown breath analyzer: Exploring correlations of breath acetone with blood glucose and glycohemoglobin A1C. IEEE Sens. J..

[30] Wang C., Surampudi A.B. (2008). An acetone breath analyzer using cavity ringdown spectroscopy: An initial test with human subjects under various situations. Meas. Sci. Technol..

[31] Yujing M., Mellouki A. (2000). The near-UV absorption cross sections for several ketones. J. Photochem. Photobio. A Chem..

[32] Da Silva F.F., Nobre M., Fernandes A., Antunes R., Almeida D., Garcia G., Mason N.J., Limão-Vieira P. (2008). Spectroscopic studies of ketones as a marker for patients with diabetes. J. Phys. Conf. Ser..

[33] NIST Chemistry WebBook Acetone UV/VIS Spectrum; Acetone IR Spectrum..

[34] Campuzano-Jost P., Williams M.B., D'Ottone L., Hynes A.J. (2004). Kinetics and mechanism of the reaction of the hydroxyl radical with h_8_-isoprene and d_8_-isoprene: Isoprene absorption cross section, rate coefficients, and the mechanism of the hydroperoxyl radical production. J. Phys. Chem. A.

[35] Martins G., Ferreira-Rodrigues A.M., Rodrigues F.N., de Souza G.G.B., Mason N.J., Eden S., Duflot D., Flament J.-P., Hoffmann S.V., Delwiche J., Hubin-Franskin M.-J., Limão-Vieira P. (2009). Valence shell electronic spectroscopy of isoprene studied by theoretical calculations and by electron scattering, photoelectron, and absolute photoabsorption measurements. Phys. Chem. Chem. Phys..

[36] O'Keefe A.O., Deacon D.A.G. (1988). Cavity ring-down optical spectrometer for absorption measurements using pulsed laser sources. Rev. Sci. Instrum..

[37] Wang C., Miller G.P., Winstead C.B., Meyers R.A. (2008). Cavity Ringdown Laser Spectroscopy. Encyclopedia of Analytical Chemistry.

[38] Berden G., Peeters R., Meijer G. (2000). Cavity ring-down spectroscopy: Experimental schemes and applications. Int. Rev. Phys. Chem..

[39] Kushch I., Arendacká B., Štolc S., Mochalski P., Filipiak W., Schwarz K., Schwentner L., Schmid A., Dzien A., Lechleitner M., Witkovský V., Miekisch W., Schubert J., Unterkofler K., Amann A. (2008). Breath isoprene—Aspects of normal physiology related to age, gender and cholesterol profile as determined in a proton transfer reaction mass spectrometry study. Clin. Chem. Lab Med..

[40] King J., Koc H., Unterkofler K., Mochalski P., Kupferthaler A., Teschl G., Teschl S., Hinterhuber H., A. A. (2010). Physiological modeling of isoprene dynamics in exhaled breath. J. Theoret. Biol..

[41] King J., Kupferthaler A., Unterkofler K., Koc H., Teschl S., Teschl G., Miekisch W., Schubert J., Hinterhuber H., Amann A. (2009). Isoprene and acetone concentration profiles during exercise at an ergometer. J. Breath Res..

[42] Lagesson V., Lagesson-Andrasko L., Andrasko J., Baco F. (2000). Identification of compounds and specific functional groups in the wavelength region 168-330 nm using gas chromatography with UV detection. J. Chromatogr. A.

[43] Kinoyanma M., Nitta H., Watanabe A., Ueda H. (2008). Acetone and isoprene concentration in exhaled breath in healthy subjects. J. Health Sci..

[44] King J., Mochalski P., Kupferthaler A., Unterkofler K., Koc H., Filipiak W., Teschl S., Hinterhuber H., Amann A. (2010). Dynamic profiles of volatile organic compounds in exhaled breath as determined by a coupled PTR-MS/GC-MS study. Physiol. Meas..

[45] Jones A.W., Lagesson V., Tagesson C. (1995). Determination of isoprene in human breath by thermal desorption gas chromatography with ultraviolet detection. J. Chromatogr. B Biomed. Appl..

[46] Zuckermann H., Schmitz B., Hass Y. (1988). Dissociation energy of an isolated triplet acetone molecule. J. Phys. Chem..

[47] Hass Y. (2004). Photochemical α-cleavage of ketones: Revisiting acetone. Photo. Chem. Photobiol. Sci..

[48] Norrish R.G.W., Crone H.G., Saltmarsh O.D. (1934). Primary photochemical reactions. Part V. The spectroscopy and photochemical decomposition of acetone. J. Chem. Soc..

[49] Nobre M., Fernandes A., da Ferreira S.F., Antunes R., Almeida D., Kokhan V., Hoffmann S.V., Mason N.J., Eden S., Limão-Vieira P. (2008). The VUV electronic spectroscopy of acetone studied by synchrotron radiation. Phys. Chem. Chem. Phys..

[50] Turner C., Španěl P., Smith D. (2006). A longitudinal study of ammonia, acetone and propanol in the exhaled breath of 30 subjects using selected ion flow tube mass spectrometry. SIFT-MS Physiol. Meas..

[51] Smith D., Španěl P., Enderby B., Lenney W., Turner C., Davies S.J. (2010). Isoprene levels in the exhaled breath of 200 healthy pupils within the age range 7-18 years studied using SIFT-MS. J. Breath Res..

